# A DUF3494 ice-binding protein with a root cap domain in a streptophyte glacier ice alga

**DOI:** 10.3389/fpls.2023.1306511

**Published:** 2024-01-05

**Authors:** Lenka Procházková, Daniel Remias, Linda Nedbalová, James A. Raymond

**Affiliations:** ^1^ Department of Ecology, Charles University, Prague, Czechia; ^2^ Department of Environment and Biodiversity, Paris Lodron University Salzburg, Salzburg, Austria; ^3^ School of Life Sciences, University of Nevada, Las Vegas, Las Vegas, NV, United States

**Keywords:** *Ancylonema nordenskioeldii*, *Ancylonema alaskanum*, ice-binding protein, streptophytes, Morteratsch Glacier, land plant terrestrialization, DUF3494, PF06830

## Abstract

Ice-binding proteins (IBPs) of the DUF3494 type have been found in many ice-associated unicellular photoautotrophs, including chlorophytes, haptophytes, diatoms and a cyanobacterium. Unrelated IBPs have been found in many land plants (streptophytes). Here we looked for IBPs in two streptophyte algae that grow only on glaciers, a group in which IBPs have not previously been examined. The two species, *Ancylonema nordenskioeldii* and *Ancylonema. alaskanum*, belong to the class Zygnematophyceae, whose members are the closest relatives to all land plants. We found that one of them, *A. nordenskioeldii*, expresses a DUF3494-type IBP that is similar to those of their chlorophyte ancestors and that has not previously been found in any streptophytes. The protein is unusual in having what appears to be a perfect array of TXT motifs that have been implicated in water or ice binding. The IBP strongly binds to ice and almost certainly has a role in mitigating the daily freeze-thaw cycles that the alga is exposed to during late summer. No IBP was found in the second species, *A. alaskanum*, which may rely more on glycerol production for its freeze-thaw tolerance. The IBP is also unusual in having a 280-residue domain with a β sandwich structure (which we designate as the DPH domain) that is characteristic of root cap proteins of land plants, and that may have a role in forming IBP oligomers. We also examined existing transcriptome data obtained from land plants to better understand the tissue and temperature dependence of expression of this domain.

## Introduction

The colonization of terrestrial habitats by plants began approximately 470-450 million years ago with the development of land-adapted streptophyte plants from their aquatic chlorophyte ancestors (Becker and Marin, 2009). This transition was accompanied by exposure to a number of abiotic stress factors such as high irradiance, lack of mineral nutrients, varying temperature, and dehydration (Rensing, 2018), which required extensive adaptations at the molecular and cellular levels (Becker and Marin, 2009). Eventually, as land plants spread into colder climates, a prominent challenge was subzero temperatures, especially freezing and thawing, which can destroy plant cell walls. Land plants have addressed this challenge by producing a number of ice-binding proteins (IBPs), ice recrystallization inhibitors and ice nucleation inhibitors that mitigate freezing injury (Bredow and Walker, 2017; Wisniewski et al., 2020). However, there is presently no information on freezing tolerance in early streptophytes, which were and are mostly unicellular algae. (Here, following Bowles (Bowles et al., 2020), we define streptophytes as land plants and their closest algal relatives, the Charophyta).

On the other hand, much is known about freezing tolerance in microscopic photoautotrophs that are commonly found in icy environments. All such microorganisms examined so far have been found to produce IBPs that prevent freeze-thaw injury. These include diatoms (bacillariophytes), chlorophytes, prasinophytes and cyanobacteria from a variety of snow and ice habitats [see Figure 6 of (Vance et al., 2019)] (Raymond and Remias, 2019; Raymond et al., 2021). Algal IBPs are limited to cold-adapted species as none have been found in any mesophilic algae whose genomes have been sequenced. All but one of the algal IBPs known so far contain a highly conserved ice-binding domain called DUF3494 (for a review, see (Vance et al., 2019). DUF3494-type IBPs are also found in bacteria, archaea and fungi. DUF3494 domains are especially common in bacteria with over 600 examples in the databases so far. This suggests that the genes evolved first in bacteria (not necessarily for ice-binding) and then spread to eukaryotic organisms (diatoms, chlorophyte algae and fungi) by horizontal gene transfer (Raymond and Kim, 2012; Vance et al., 2019).

**Figure 1:**
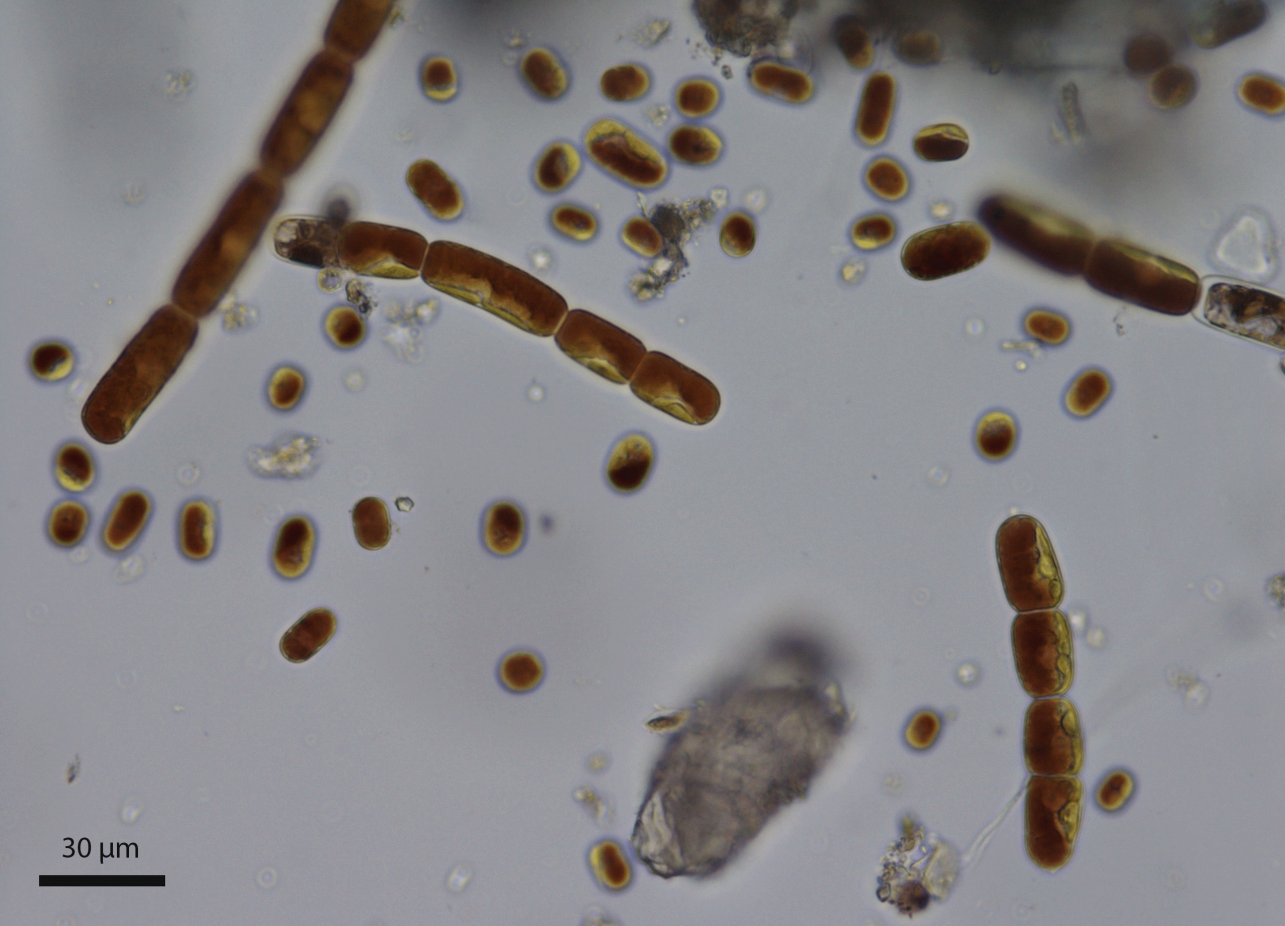
Mixture of chain-forming *Ancylonema nordenskioeldii* and the single-celled *Ancylonema alaskanum* collected in August 2020 from the Morteratsch Glacier, Switzerland (sample WP249).

A good place to look for IBPs in early streptophyte algae are the Zygnematophyceae, the closest known relatives of all land plants (Cheng et al., 2019). Two ice-associated algae in this group are the chain-forming *Ancylonema nordenskioeldii* and the essentially single-celled *A. alaskanum* (formerly *Mesotaenium berggrenii*) (**Figure 1**), which are found on glaciers. Their vacuoles are darkly pigmented with polyphenols, probably for protection from intense radiation and possibly for repelling grazers (Remias et al., 2012). (During the course of this study, an obstacle to working with these species was that they could not be cultured (Remias et al., 2012) and so their study depended on the luck of finding nearly unialgal populations in the wild. Very recently, however, methods were developed for culturing *A. alaskanum* (Remias and Procházková, 2023) and *Ancylonema* spp. (Jensen et al., 2023). Thus, if these species have IBPs, their IBPs might provide insights into the development of IBPs in streptophytes: are they the forerunners of the IBPs of land plants or similar to those of their chlorophyte ancestors? To answer this question, we examined these two species for IBPs through activity assays, ice-affinity purification, gel electrophoresis and genome and transcriptome sequencing. We found strong evidence that one of these species produces a DUF3494-type IBP and confirmed its ice-binding activity.

Furthermore, it has a streptophyte-specific C-terminal domain (here designated as the DPH domain) that is characteristic of root cap proteins of land plants and that may have a role in increasing the protein’s ice-binding activity.

## Methods

### Algae

**Figure 2:**
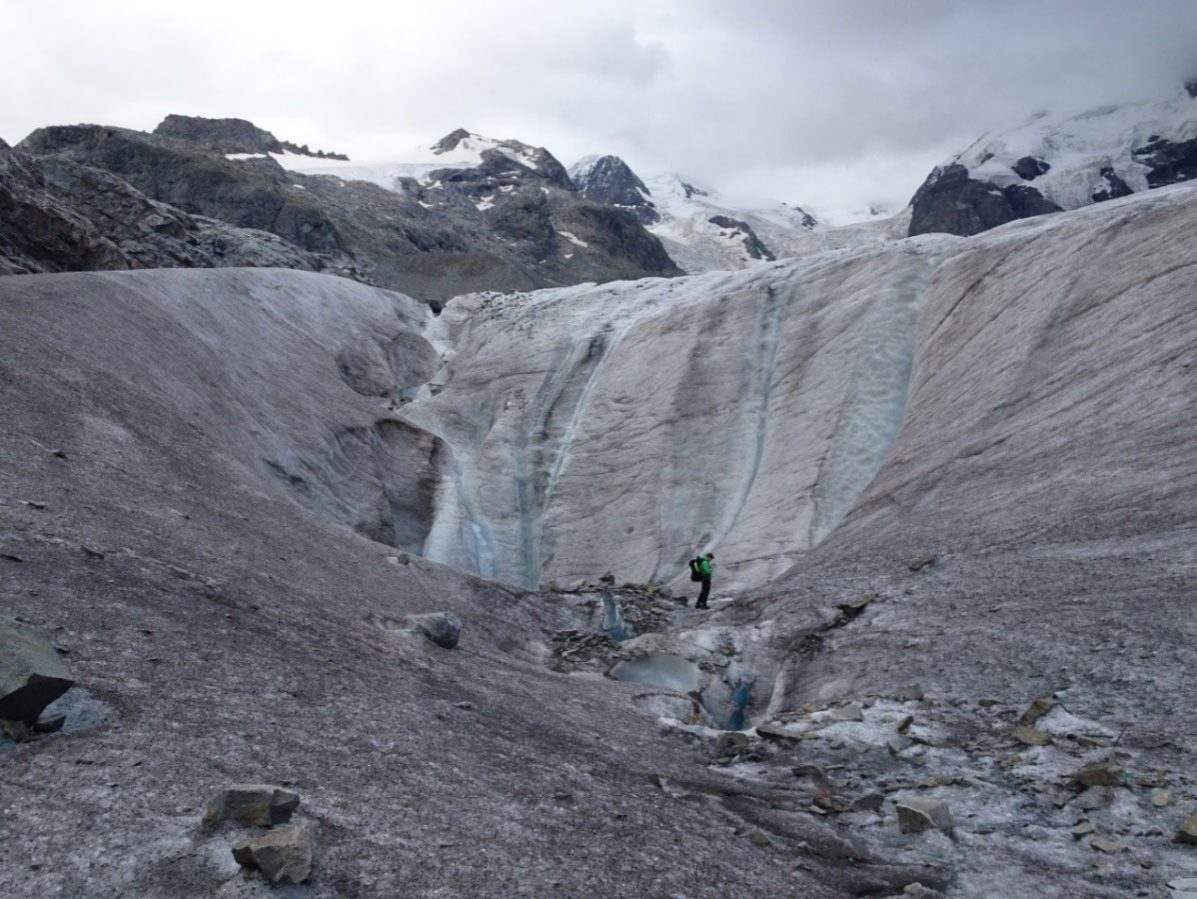
Morteratsch Glacier, Switzerland on August 25, 2021. The pinkish gray material on the ice is mainly *A. alaskanum* and partly *A. nordenskioeldii*. A team member provides scale.

Surface ice samples were collected from the Morteratsch Glacier in Switzerland (46.40° N, 9.93° E) (**Figure 2**) and the Gurgler Glacier in Austria (46.80° N, 10.98° E) between 2018 and 2021 as described previously (Procházková et al., 2021) (**Table 1**). Collections were made at the end of season in the Alps, in late August when algal biomass was at a maximum. Briefly, samples were identified with a field microscope and collected with tools sterilized with 70% ethanol. The ice was gently melted in the dark at 4°C overnight. Larger rock debris was removed by sieving the meltwater through a sieve tower (final mesh 140 µm) (Retsch, Germany). WP274 cells, from which RNA was extracted, were then kept at 1°C for 5 h at about 80–150 µmol photosynthetically active radiation (PAR) under laboratory conditions and then mixed with an equal volume of RNAlater (Ambion). RNA was extracted from the RNAlater within 24 hours. Light microscopy was performed with an Olympus BX43 at 1000× magnification.

**Table 1:** Collections of *Ancylonema* spp. (Zygnematophyceae) used in this study.

Date	Isolate	Glacier	Species ratio^1^	IBP activity	Sequen- cing
Aug 22, 2018	WP211	Morteratsch	An	+	DNA
Aug 17, 2020	WP249	Morteratsch	An : Aa ~1:1	+	
Aug 25, 2020	WP251	Gurgler Ferner	S:Aa <1:20	-	
Aug 25, 2021	WP274	Morteratsch	An : Aa <1:20	-	RNA

^1^An, *A. nordenskioeldii*; Aa, *A. alaskanum*; S, *Sanguina nivaloides* (Chlorophyceae).

### Ice-binding protein activity and purification

Subsamples WP211, WP249, WP251 and WP274 were lyophilized overnight and shipped to University of Nevada Las Vegas. Ice-binding activity was estimated by observing irregularities in the surface of a slowly growing ice seed crystal submerged in the supernatant of cells ground in water with a mortar and pestle (Raymond and Fritsen, 2000). Seed crystals were perfect, plate-like crystals of approximately 5 mm diameter, in which the ice *c*-axis (the axis normal to a snowflake) was normal to the flat surface (basal plane). Semi-purified IBP was obtained by ice-affinity purification, consisting of several cycles of freezing with 150 mM NaCl, centrifugation at -5°C, and thawing, as described previously (Raymond and Fritsen, 2001).

### 2D electrophoresis

Ice-affinity purified samples were freeze dried and sent to Kendrick Laboratories (Madison, WI, USA) for 2D electrophoresis with syproruby staining. Electrophoresis was performed according to the carrier ampholine method of isoelectric focusing (Kendrick et al., 2020). Isoelectric focusing was carried out in a glass tube of inner diameter 3.3 mm using 2.0% pH 3-10 Isodalt Servalytes (Serva, Heidelberg, Germany) for 20,000 volt-hrs. One µg of an IEF internal standard, tropomyosin, was added to each sample. After equilibration for 10 min in buffer “O” (10% glycerol, 50 mM dithiothreitol, 2.3% SDS and 0.0625 M tris, pH 6.8), each tube gel was sealed to the top of a stacking gel that overlaid a 10% acrylamide slab gel (1.0 mm thick). SDS slab gel electrophoresis was carried out for about 5 hrs at 25 mA/gel. The following proteins (Millipore Sigma) were used as molecular weight standards: myosin (220,000), phosphorylase A (94,000), catalase (60,000), actin (43,000), carbonic anhydrase (29,000), and lysozyme (14,000).

### DNA and RNA sequencing

The WP211 and WP274 samples were considered as environmental samples as they contained other microorganisms present in the ice, although under the microscope, virtually all the biomass appeared to be due to *Ancylonema* spp.

WP211 cells were mechanically disrupted by shaking for 5 min (30 Hz) in the presence of 3-mm glass beads (Sigma–Aldrich) in an MM 400 Mixer Mill (Retsch, Germany). DNA was prepared from the disrupted cells with a DNeasy Plant Mini Kit (Qiagen, Germany) and sent to Seqomics Biotechnology Ltd. (Morahalom, Hungary) for library preparation and sequencing. The DNA library was prepared with a NEBNext II DNA kit (Illumina) and sequenced with Illumina MiSeq mate-paired sequencing. The WP211 sample yielded 13,342,240 quality filtered reads of 250 nt each. A Kraken2 analysis (Wood and Salzberg, 2014) by Seqomics showed that a large fraction of the reads could be classified as bacterial reads. The reads were binned into bacterial and algal bins and the algal reads were assembled with MetaEuk (Levy Karin et al., 2020) into 7425 contigs with a total length of 42,187,391 bp.

WP274 RNA was prepared from the RNAlater WP274 sample with an E.Z.N.A. Plant RNA Kit (Omega Bio-Tek, Georgia) using the difficult samples protocol and sent to Seqomics for library preparation and sequencing. The RNA library was prepared with a Zymo-Seq Ribofree Total RNA Library Kit (Zymo Research) and sequenced with Illumina NextSeq mate-paired sequencing, yielding 25,048,640 quality filtered reads of 150 nt each. A Kraken2 analysis by Seqomics showed that many of the reads could be classified as bacterial reads. The reads were binned into bacterial and algal bins and the algal reads were assembled with MetaEuk into 54,541 contigs with a total length of 24,335,360 bp.

### 3D structure prediction

The structure of *A. nordenskioldii* IBP protein (WHL30856.1; assembled from the WP211 metagenome) was predicted with the ColabFold notebook (Mirdita et al., 2022) which is based on AlphaFold21 (Jumper et al., 2021) and implemented by UCSF ChimeraX v. 1.6rc (Pettersen et al., 2021) using the default parameters. An N-terminal signal peptide was identified with SignalP 5.0 (https://services.healthtech.dtu.dk/services/SignalP-5.0/). Molecular weight and pI were calculated with an Expasy tool (https://web.expasy.org/compute_pi/). The predicted protein structure was viewed with the Yasara viewer (Krieger and Vriend, 2014).

### Classification of DPH domains

In this study, the DPH domain is defined as a ~280-residue C-terminal sequence starting with DPH (or DPR in grasses) and including a ~60-residue root cap domain at its C-terminus. A search for DPH-like sequences in NCBI’s non-redundant (nr) database yielded over 2,000 hits with evalues <1e-10. All the sequences were determined to be streptophyte sequences by using a custom script written in BioPython (available on request).

### DPH expression in grass tissues

DPH expression in different tissues and at different temperatures was analyzed in publicly available RNA-seq data. DPH domains (which begin with DPR in grasses) were obtained for each grass by protein blasts using *An*IBP’s DPH domain as a query. The nucleotide sequences of the hits were obtained and the portion upstream of the sequence encoding DPR was deleted. These sequences were then used as queries in blasts of the RNA-seq data in NCBI’s SRA database. Expression of the DPH domains was expressed as the number of unique hits per million reads of the transcriptome, or as FPKM values (fragments per kilobase of query per million reads). In the grass tissue analysis, the values for the different DPHs were averaged. The BioProjects and DPH queries used for each species are shown in **Supplementary Table S1** (grass tissue) and **Supplementary Table S2** (temperature).

## Results

### Algae identification

The algae in the WP211 sample were microscopically identified as almost entirely *A. nordenskioeldii*. They could be distinguished from *A. alaskanum* cells by their larger size and chain formation (**Figure 1**). Although *A. nordenskioeldii*. appeared to account for almost all the biomass in the sample, a Kraken analysis showed that a substantial fraction of the DNA reads was of bacterial origin. One prominent 18S rRNA sequence was assembled (acc. no. OQ642151) and it was identical to the sequence of a sample of *A. nordenskioeldii* collected in Svalbard (AF514397.2). The algae in the WP274 sample were microscopically identified as mostly *A. alaskanum* with a small percentage of *A. nordenskioeldii* (**Table 1**). An 18S rRNA gene sequence was assembled from transcripts (acc. no. OQ642152) and was found to be identical to that of an *A. alaskanum* sample obtained from an Austrian glacier (JF430424.1). The 18S sequences of the two species are nearly (99.7%) identical, with short variable regions.

### Ice-binding protein activity

**Figure 3:**
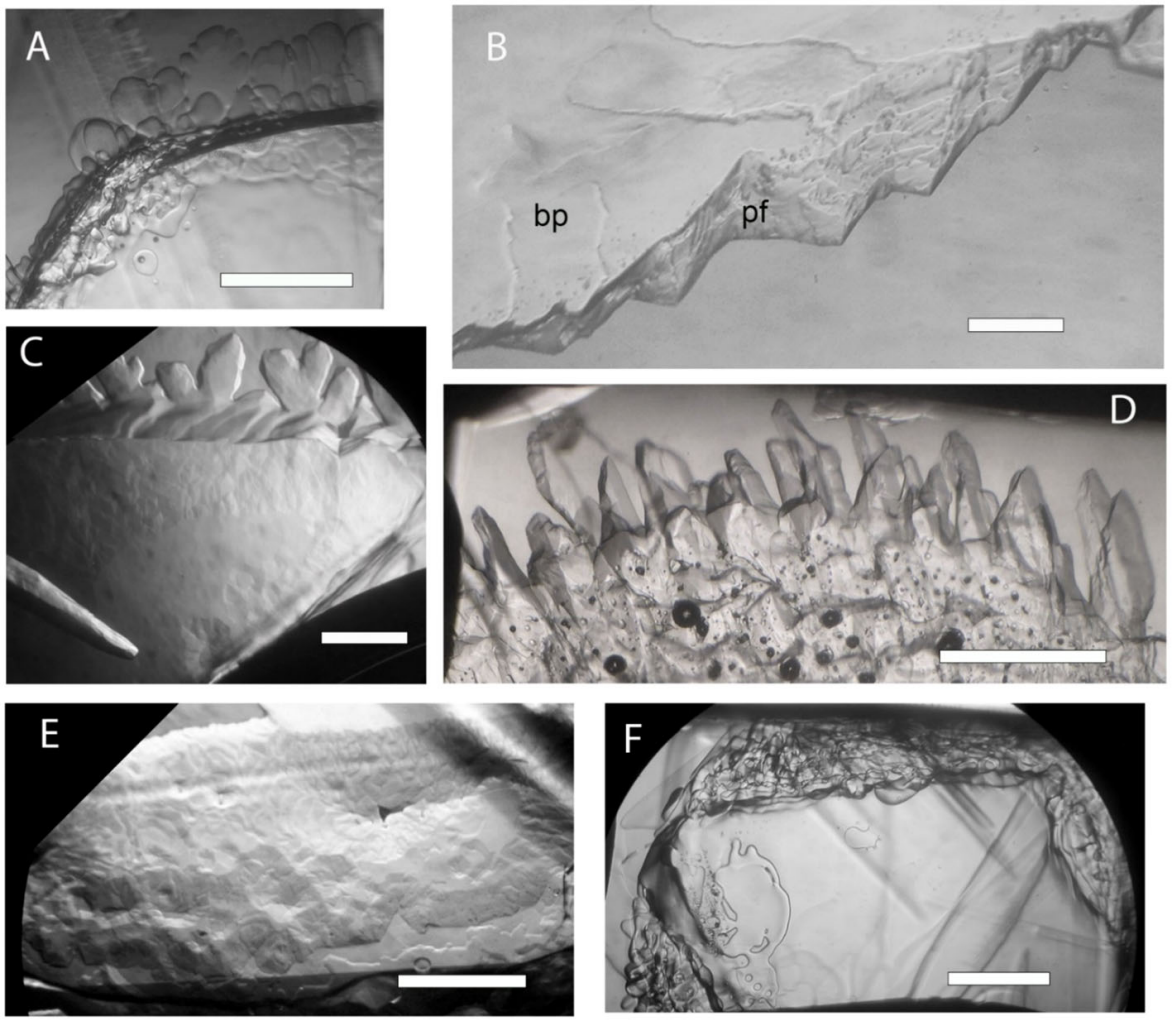
Growth of perfect ice crystals submerged in *Ancylonema* cell homogenate supernatants. The views are along the ice c-axis onto the basal plane except in B where the basal plane is tilted up to better show the prism face. The ice crystals were allowed to grow at just below the freezing point for 10 to 60 min. **(A)**, No activity was observed in a sample consisting of almost entirely of *A. alaskanum* (WP274), as shown by rounded dendrites and smooth basal plane. **(B)**, Strongly inhibited growth on basal plane (bp) and prism face (pf) in presence of *A. nordenskioeldii* (WP211). **(C, D)**, Faceted growth and mottled basal plane after 2 cycles **(C)** and six cycles **(D)** of ice affinity purification of *A. nordenskioeldii* supernatant. **(E, F)**, Approximately equal mixture of *A. nordenskioeldii* and *A. alaskanum* (sample WP249). Strong activity as shown by the pitted basal plane **(E)** was destroyed by 10 min at 65°C **(F)**. Scale bars, 1 mm.

Four samples were checked for IBP activity (**Table 1**). No IBP activity was observed in the WP274 sample (almost all *A. alaskanum*) (**Figure 3A**) or the WP251 sample (also almost all *A. alaskanum*) (data not shown), suggesting that *A. alaskanum* does not produce an IBP. However, cell homogenate supernatants of *A. nordenskioeldii* showed clear ice-structuring activity, in which growth was inhibited on both the ice basal plane and prism faces (**Figure 3B**). The activity (shown by faceted growth and pitted basal plane) was retained after subjecting the homogenate to 2 and 6 cycles of ice-affinity purification (**Figures 3C, D**, respectively), confirming the protein’s affinity for ice. Another sample that contained both *Ancylonema* species (WP249) showed strong activity (**Figure 3E**), which was attributed to *A. nordenskioeldii* because the *A. alaskanum* samples were inactive. Heating the sample shown in **Figure 3E** to 65°C for 10 min destroyed its activity (**Figure 3F**), which strongly suggested that the active substance was one or more proteins.

### IBP identity

A search for known plant ice-binding proteins (Bredow and Walker, 2017) in the WP211 DNA sequence data (which contain only *A. nordenskioeldii* sequences, **Table 1**) yielded only DUF3494-type sequences, suggesting that they were responsible for the observed activity. A total of 201 such sequences were found, 188 of which could be attributed to a single gene encoded by one of the contigs, K141_331835. It was very likely an algal contig because the contigs were assembled from binned algal reads. The remaining 13 reads were attributed to bacterial IBPs in the metagenome, suggesting that most of the observed IBP activity was due to the gene in contig K141_331835. The source of this gene could not be immediately identified because its C-terminal region could not be translated. However, sequence data recently submitted to GenBank (Sekimoto et al., 2023) made it possible to identify three introns in the C-terminal region and thus obtain its sequence. The new data was from two isolates of the closely related alga *Closterium* (Zygnematophyceae) obtained from soil and water samples in Nepal. The *Closterium* sequences (e.g., GJP46862.1) were also very close (e-values <1e-82) to the C-terminal domain of the gene in contig K141_331835. This clearly identified the DUF3494 gene in contig K141_331835I as belonging to *A. nordenskioeldii*. The C-terminal domain begins immediately after the IBP domain with the sequence DPH (Asp-Pro-His) and has a length of 281 a.a. As the *Closterium* sequences also begin with DPH, we designate this as the DPH domain.

**Figure 4:**
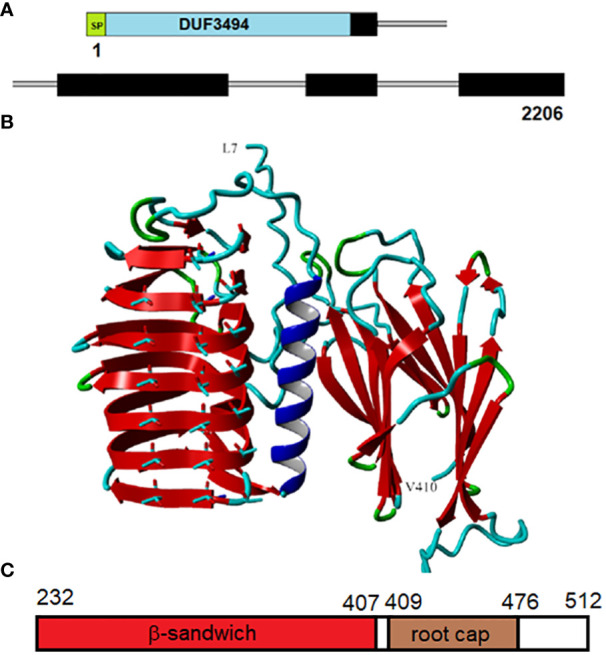
Structure of *An*IBP. **(A)**, Structure of the 2206-nt gene (OQ653557.1). The domains include a signal peptide (SP), DUF3494 domain (blue), and *Closterium*-like C-terminal region (black), designated the DPH domain (which starts with DPH). Introns are shown by thin gray lines. **(B)**, Predicted structure of the protein from L7 to V410. The region beyond V410 could not be accurately modeled. The DUF3494 domain with its β−solenoid and prominent alpha helix is on the left. The predicted ice-binding side with three rows of aligned Threonyl residues is facing forward. The DPH domain is on the right and is predicted to form two parallel β−sheets called a β−sandwich. The view is through the space between the two sheets. Color codes: red, β−sheet; blue, alpha helix; cyan, coil; green, turn. A movie of the rotating protein is shown in Supplementary Movie SM1. **(C)**, Structure of the DPH domain. The domain includes a β sandwich followed by a root cap domain. Residue positions are shown at the top.

The IBP gene (OQ653557) has a length of 2206 bp encoding four exons, with all of the introns in the DPH domain (**Figure 4A**). The protein (WHL30856.1), which we designate as *An*IBP, has a length of 515 a.a. residues encoding a predicted 18-a.a. N-terminal signal peptide, a DUF3494 domain and the DPH domain with a predicted molecular mass of 54.5 kDa (with signal peptide) and a predicted pI of 5.4.

**Figure 5:**
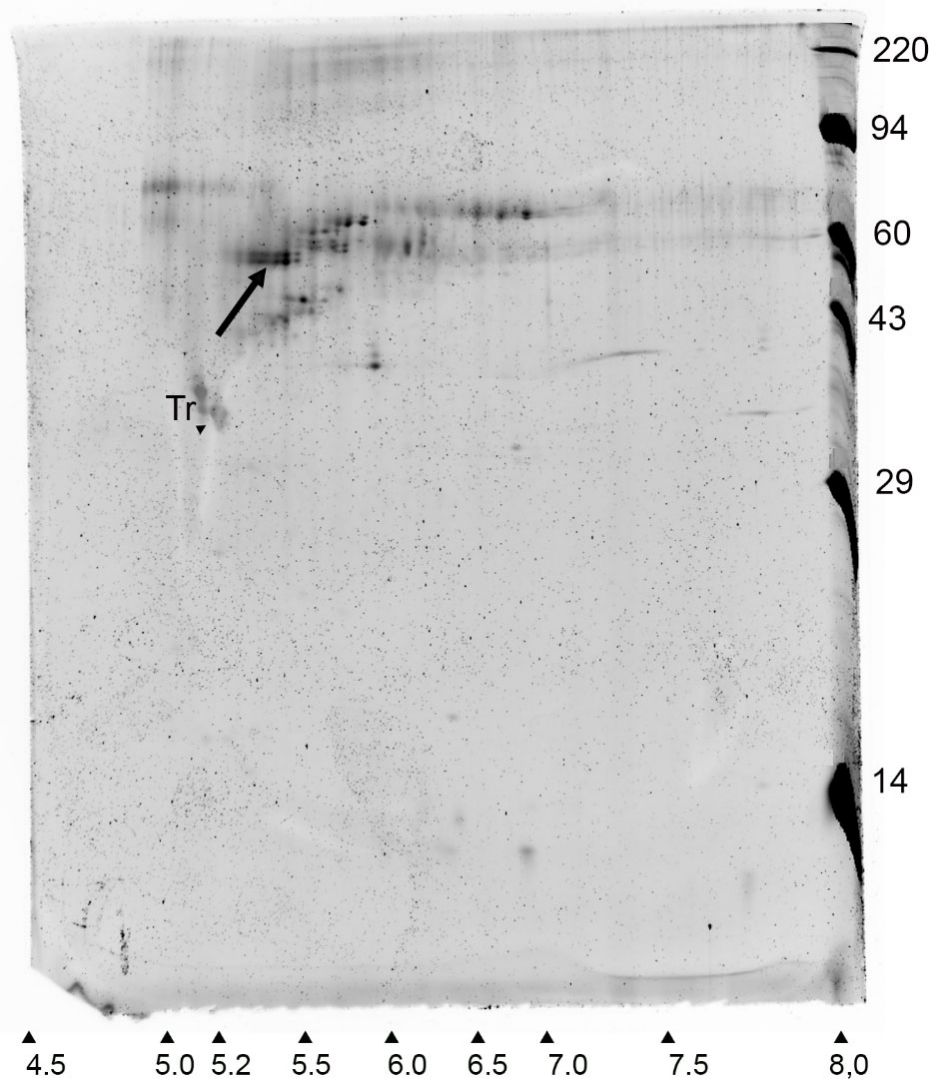
Syproruby-stained 2D electrophoretic gel of an ice affinity-purified sample of *A. nordenskioeldii* IBP. Arrow indicates a candidate IBP. Horizontal and vertical axes show pI and MW in kDa, respectively. Tr, tropomyosin internal standard.

A WP211 *A. nordenskioeldii* sample semipurified by two cycles of ice affinity purification was freeze dried and subjected to 2D electrophoresis. The gel contained a prominent spot with a molecular mass of approximately 55 kDa and a pI of approximately 5.4 (**Figure 5**), closely resembling the predicted MW and pI of *An*IBP. Similarly acidic pIs have been found in other IBPs (Janech et al., 2006; Raymond et al., 2007; Raymond et al., 2009). Attempts to identify peptides in the gel spots by mass spectrometry were unsuccessful. However, the close matching of the gel spot with the predicted mass and pI of *An*IBP and the fact that the spot had gone through two cycles of ice-affinity purification confirms that *An*IBP is an ice-binding protein.

This is the first time a DUF3494-type IBP has been found in a streptophyte. The DUF3494 region did not closely match those of other DUF3494 proteins, with the closest matches being fungal and bacterial proteins with about 48% and 45% amino acid sequence identities, respectively. Searches of the WP251 and WP274 transcriptomes (both almost entirely consisting of *A. alaskanum*) did not find any genes encoding an *An*IBP-like protein, in agreement with the negative IBP activity assays of these samples (**Figure 3A**).

### Predicted *An*IBP structure

*An*IBP’s structure, as predicted by AlphaFold, is shown in **Figure 4B**. AlphaFold predicted high accuracies (i.e., low pLDDT values) for all the β−strand structures shown. Regions of lower accuracy (residues 1-6 and 411-512) are not shown. A movie of the rotating protein is shown in **Supplementary Movie SM1**. The DUF3494 domain was predicted to have a structure like those of other DUF3494 proteins: a triagonal β−fold solenoid consisting of 7 discontinuous coils (order of coils in **Figure 4B** from top to bottom is 3,4,5,6,7,1,2 with an alpha helical region between coils 2 and 3. The side of the solenoid that has usually been implicated in ice binding has three well-aligned rows of Thr residues, two of which form a row of TXT motifs, where X is any amino acid. TXT motifs have been implicated in ice binding in several IBPs including insect antifreeze proteins (see below). The perfect alignment of the Thr residues supports the accuracy of the DUF3494 portion of the model.

*An*IBP’s DPH domain was found to have two subdomains, an N-terminal portion predicted by AlphaFold to form a β sandwich with high confidence, followed by a ~60-residue domain identified by NCBI’s conserved domain database as “root cap” (**Figure 4C**). β Sandwiches form a large family of proteins, many of which are associated with adhesion (Vance et al., 2018). The root cap domain is found in many root cap proteins of land plants. The DPH domain including root cap has previously been found in other streptophytes (see Discussion). The β sandwich (**Figure 4B**) consists of two parallel β sheets, each consisting of four β strands. Its orientation with respect to the DUF3494 domain is unclear as some predictions show it rotated forward 90 degrees so that the β−strands in the two domains are more or less on the same horizontal plane. However, in none of the predictions does the DPH domain block the ice-binding side of the DUF3494 domain.

### Occurrence of DPH domains

Blasting the *An*IBP DPH domain against the NCBI nr database yielded over 2,100 hits with evalues <1e-10, representing 199 genera. All of the genera were determined to be streptophytes from a wide variety of classes, including Zygnematophyceae, Poaceae, ferns and charophytes (see Methods). Additionally, the genomes of many species have many such proteins; *Closterium* sp. NIES68 and *Triticum aestivum* have at least 50 of them. Together, these results suggest that the DPH domain has an important role in streptophytes.

To check the root specificity of the DPH domain in land plants, we examined some recent RNA-seq studies that obtained transcriptomes from different tissues in grasses. In agreement with the results of Matsuyama et al., DPH domains were most strongly expressed in root tissue and weakly expressed in leaf, spike and stem tissues (**Table 2**).

**Table 2:** Expression of DPH domains in different tissues of grasses.

Species	non-root tissue		root
*Lolium perenne*	stem	0.23	44.41
*Panicum miliaceum*	leaf	0.02	20.29
*Triticum aestivum*	leaf	0.01	18.72
*Hordeum vulgare1*	leaf	0.00	10.22
*Hordeum vulgare2*	leaf	0.03	9.61
*Oryza sativa*	leaf	0.86	3.70
*Triticum turgidum durum*	leaf-stem	0.01	1.48
	flagleaf	0.01	
	spike	0.04	

The values indicate the average number of hits for 3 to 6 DPH domains per RNA-seq experiment per million reads. These data are condensed from original the data shown in **Supplementary Table S1**.

## Discussion

### IBP domain

The preceding results demonstrate that a basal streptophyte, one belonging to the class Zygnematophyceae, acquired a DUF3494-type ice-binding protein similar to those in chlorophytes, bacteria and fungi. Land plants have developed a number of non-DUF3494-type IBPs (including ice-recrystallization inhibiting proteins), that fall into two major groups, pathogen-related proteins (Bredow and Walker, 2017) and left-handed β−roll structures that are found in several grasses (John et al., 2009; Middleton et al., 2012; Sformo and Raymond, 2020). The DUF3494 IBP of *A. nordenskioeldii* thus appears to lack a homolog in any land plants.

The *An*IBP DUF3494 domain stands out compared to other DUF3494 IBPs in its high number of regularly spaced TXT motifs, where X is any amino acid. None of the DUF3494 structures that have been determined so far (Vance 2019) show such an orderly array of TXT motifs. These motifs have an affinity for water molecules and have been implicated in ice-binding in the grass *Lolium perenne* (Kuiper et al., 2001), insect antifreeze proteins (Graether and Sykes, 2004) and an algal IBP (Raymond et al., 2009). Water molecules bound to the TXT motifs may serve as a glue that binds the protein to an ice lattice. The average distance between the coils in the model is about 5.0 Å, which is not too different from the ice a-axis repeat distance, 4.52 Å. The N-terminal signal peptide could function as either a secretion signal (in which case it is normally cleaved during secretion) or it could remain intact and anchor the IBP to the cell membrane, where it might also function extracellularly. The latter seems more likely because of the close match of the observed molecular weight (**Figure 5**) with the predicted value. In either case, it could prevent the recrystallization of extracellular ice during freeze-thaw cycles, which is considered an important factor in freezing tolerance (Zhang et al., 2010; Bredow et al., 2016).

The origin of the *Ancylonema* IBP is unclear. Blast searches found no other DUF3494 proteins in the Charophyceae, which comprise the early streptophytes. Although no close matches to other microbial IBPs were found, that does not rule out the possibility that the gene was acquired by horizontal gene transfer from bacteria, as has been proposed for other algal IBPs (Raymond and Kim, 2012; Vance et al., 2019). The absence of introns in the DUF3494 domain of *An*IBP is consistent with such a transfer. Indeed, some of the key genes that allowed the Zygnematophyceae to survive desiccation and other stresses on land were mostly likely acquired from soil bacteria (Cheng et al., 2019).

Curiously, no IBP homolog or IBP activity was found in *A. alaskanum*, as all other ice-associated microalgae that have been examined so far were found to have some type of IBP. So, *A. alaskanum* must have some other mechanisms for avoiding freeze-thaw injuries. At least part of its freeze-thaw tolerance probably comes from glycerol production, as glycerol was found to account for 11% of its total soluble carbohydrates (Roser et al., 1992) and was one to two orders of magnitude greater in abundance than other sugars and sugar alcohols (Wastian, 2010). This and other adaptations may have also contributed to the loss of an IBP similar to *An*IBP in the past (gene loss in algae is well documented [e.g., (Bowles et al., 2020)]. This difference between *A. alaskanum* and *A. nordenskioeldii* may partially account for their different distribution patterns, with the former dominating at Alpine and Pacific (humid) sites and the latter dominating in the drier High Arctic (Procházková et al., 2021; Remias et al., 2023).

### DPH domain

Land plants have an organ at the tip of their roots called a root cap, or calyptra, that protects the stem cells at the root tip and that receives and transmits environmental signals to the growing root (Kumpf and Nowack, 2015). The root tip was first noticed by Charles Darwin in 1880, who concluded that it had great importance in determining root growth as well as development of the entire plant (Kumpf and Nowack, 2015). The root cap has been designated as protein family PF06380 (https://www.ebi.ac.uk/interpro/entry/pfam/PF06830/), which describes it as a ~60 residue domain found in many root cap proteins. The conserved region was first pointed out by Matsuyama et al. (Matsuyama et al., 1999) who showed that it was specifically expressed in the root caps of maize, white spruce, and Arabidopsis, but that it was much larger than the 60-residue pfam domain (and very similar to *An*IBP’s DPH domain). Nedelcu et al. (Nedelcu et al., 2006) found a similar domain (also starting with DPH) in the freshwater unicellular alga *Mesostigma viride*, which they used to classify *M. viride* as a streptophyte.

It should be pointed out that another type of β sandwich, called bacterial immunoglobulin domains (Bigs), are found with bacterial DUF3494-type IBPs (Vance et al., 2018). They typically occur in tandem strings upstream of bacterial DUF3494 domains. Big_5 and Big_2 are the most common types; GenBank has over 400 DUF3494 IBPs with Big_5 domains. In Bigs, the sandwich consists of only two antiparallel β strands whereas in the DPH domains, it consists of two β sheets, each consisting of four β strands. The function of the Bigs is unclear but they have been suggested to act as ice adhesins (Vance et al., 2018). It is unclear whether their role is related to the role of the DPH domain in *An*IBP.

In agreement with our DPH blast results, Pfam lists about 2,000 PF06830 proteins (https://www.ebi.ac.uk/interpro/entry/InterPro/IPR009646/protein/UniProt/#table), as well as shows their AlphaFold-predicted structures. Random sampling of these proteins suggests that they all contain a DPH domain dominated by a β sandwich structure as well as a root cap domain at the C-terminus. However, to the best of our knowledge, the present report is the first to explicitly state that this region forms a β sandwich. In view of the wide occurrence of these proteins, it is surprising that they have received so little attention in the literature. In land plants, they may be involved in the major functions of the root cap, such as protection of the root tip and sensing and transmitting environmental signals, while in streptophyte algae (e.g., *Ancylonema* and *Closterium*) they may have related roles or other unrelated roles.

**Figure 6:**
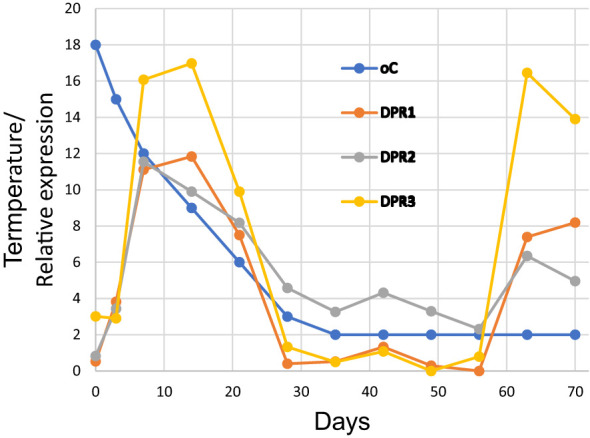
Expression of three DPR domains of *Triticum aestivum* during cold adaptation over a 70-day period. The underlying data are shown in **Supplementary Table S2** and were obtained from BioProject PRJNA564622.

One approach to understanding the function of the DPH domains in land plants is to look at existing RNA-seq data to see under what conditions the DPH domains are expressed. In one experiment (Rabanus-Wallace et al., 2021), *Triticum aestivum* (whose DPH domains begin with DPR) was cooled from 18°C to 2°C over a 70-day period and root transcriptomes were obtained at regular intervals. We randomly selected three of *T. aestivum*’s ~50 DPR domains and looked at their expressions during the cooling period (**Figure 6**). The expressions were very similar, each showing two major peaks, first on about day 10 when the temperature had decreased to about 0°C, and then again on about day 63 after about 4 weeks at 2°C. These results appear to show that the DPH domains have a role in *T. aestivum*’s cold response.

It is interesting that PF06830 proteins share several characteristics with small heat shock proteins (sHSPs), including high abundance and diversity, small size (<25 kDa), β sandwich structures, C-terminal positions and variable N-terminal regions (Waters and Vierling, 2020). sHSPs in plants as well as other organisms are upregulated in response to abiotic stresses and have been proposed to act as chaperones that prevent damage to proteins by such stresses (Haslbeck and Vierling, 2015; Waters and Vierling, 2020). This raises the possibility that PF06830 proteins carry out their protective functions as sHSPs, although there is presently no direct evidence for this. Another characteristic of sHSPs is that they tend to assemble into larger oligomers due to the adhesive properties of the β sandwiches (Basha et al., 2012). In the case of *An*IBP, this could possibly result in the formation of IBP oligomers with increased ice-binding activity.

In summary, we describe a protein in a glacier ice alga that is probably essential for its survival and that illustrates one of the perils (freeze-thaw exposure) that early land plants had to face to gain a foothold on land. The protein is interesting in having a chlorophyte-like ice-binding domain unlike the ice-binding domains of land plants, and a second (DPH) domain that in some way enhances its protective ability, possibly by inducing the formation of IBP oligomers. Our results also bring needed attention to the great abundance and diversity of root cap proteins, whose functions are only beginning to be understood.

## Data availability statement

The original contributions presented in the study are publicly available. This data can be found here: https://www.ncbi.nlm.nih.gov/genbank/, OQ653557; https://www.ncbi.nlm.nih.gov/genbank/, OQ642151.

## Author contributions

LP: Conceptualization, Investigation, Methodology, Resources, Visualization, Writing – review & editing. DR: Conceptualization, Funding acquisition, Investigation, Methodology, Resources, Writing – review & editing, Validation. LN: Funding acquisition, Resources, Writing – review & editing, Formal Analysis. JR: Conceptualization, Investigation, Methodology, Resources, Visualization, Writing – original draft, Writing – review & editing.
